# Quantitative Mapping of the Lipid Nanoenvironment around Transmembrane Proteins in Living Cells

**DOI:** 10.1021/acsnano.5c19300

**Published:** 2026-01-10

**Authors:** Veronika Brumovska, Marina Bishara, Andreas M. Arnold, Barbora Kalouskova, Gergö Fülöp, Marc Fahrner, Isabella Derler, Lena Maltan, Nobuaki Matsumori, Mario Brameshuber, Gerhard J. Schütz, Eva Sevcsik

**Affiliations:** † Institute of Applied Physics, TU Wien, Wiedner Hauptstr. 8-10, 1040 Vienna, Austria; ‡ Biochemistry & Biophysics Center, National Heart, Lung, and Blood Institute, National Institutes of Health, 50 South Drive, Bethesda, Maryland 20892, United States; § Institute of Biophysics, JKU Life Science Center, Johannes Kepler University Linz, Gruberstraße 40, 4020 Linz, Austria; ∥ Department of Chemistry, Graduate School of Science, Kyushu University, Fukuoka 819-0395, Japan

**Keywords:** plasma membrane organization, transmembrane protein, lipid−protein interaction, protein nanoenvironment, single molecule tracking

## Abstract

Weak and transient lipid–protein interactions are thought to shape plasma membrane organization and function but have largely eluded experimental characterization. While model systems can only capture certain aspects of these interactions, extraction of unambiguous data from live cell experiments is challenging. We here ask a simple question directed at a fundamental aspect of plasma membrane organization: To what extent does a transmembrane protein influence, by its mere presence, the fluidity of its immediate lipid nanoenvironment? By specifically immobilizing proteins of interest at various densities in the live cell plasma membrane, we were able to determine its apparent in-plane hydrodynamic radius via quantification of the mobility reduction of individual lipid tracer molecules. In this assay, tight adhesion of lipid layers with reduced fluidity would manifest as an increased effective protein radius. We compared these values with structural biology data and used simulations to map the parameter space of possible nanoenvironment architectures around four different transmembrane proteins. For three of the four proteins tested, our data rule out the presence of tightly associated boundary lipids, calling into question their role as a general membrane-organizing principle.

## Graphical abstract

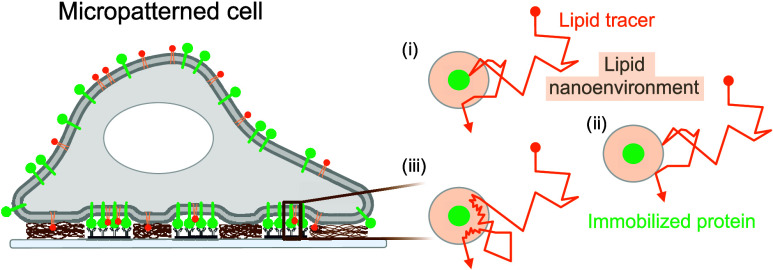

Plasma membrane function and organization are mediated by the dynamic and intricate interplay of thousands of different proteins and lipids. Lipids assume diverse roles in plasma membrane organization: As building blocks of the membrane, they provide a matrix for transmembrane proteins and collectively create a membrane environment characterized by, among others, a certain lateral pressure profile, membrane thickness and charge distribution, which can shape protein function.^1,2^ In addition, individual lipids have been shown to modulate protein conformation and function by specific binding with affinities that in some cases even allow cocrystallization of the bound lipid along with the protein.^3–5^ In between these two extremes fall a cornucopia of lipid–protein interactions with a wide range of specificities and affinities which have been proposed to act concertedly to create an idiosyncratic lipid nanoenvironment around each transmembrane protein,^1,2,6,7^ thereby promoting optimum functionality. Whether all transmembrane proteins feature such nanoenvironments, and what their qualities are, is largely unknown.

Envisioning a specific transmembrane protein, it seems plausible that a lipid in direct contact with the protein surface has different characteristics than a bulk lipid, or a lipid in contact with a different protein. The protein’s specific structure with its distribution of charged residues,^8^ its surface area^9^ and roughness,^10^ the length of its transmembrane region and/or its tilt versus the membrane normal^9,11^ may favor the association of particular lipid species and exclude others, and may as well influence the properties of those lipids that are in direct contact with the protein surface (e.g., chain order, packing parameter, tilt^11^). The concept of “boundary” or “annular” lipids associated with transmembrane proteins has been proposed several decades ago, based on electron spin resonance (ESR) experiments in reconstituted lipid/protein systems that revealed a lipid subpopulation with lower mobility.^12^ Indeed, native mass spectrometry has identified association of lipids with a number of proteins at different levels of selectivity.^3,4,13–16^ Apart from modulating protein function, these tightly associated lipids may then in turn shape the lipid nanoenvironment around the transmembrane protein.^1,7,17^ A more lipid-centric concept of a lipid nanoenvironment has been introduced by the raft hypothesis, which postulates that sterol-dependent nanoscopic membrane domains of higher viscosity and order compartmentalize proteins.^18^ In this view, proteins would, according to their raft affinity, be surrounded by a more or less ordered lipid nanoenvironment,^9^ and even modulate their nanoenvironment as a response to e.g., changes in conformation or oligomeric state, with consequences for protein function. On the other hand, protein reorganization as it occurs in the course of signaling events may alter the lipid nanoenvironment, in turn influencing the signaling response.^19^

When attempting to study the lipid nanoenvironment of a transmembrane protein, one is faced with a conundrum: Techniques such as native mass spectrometry, crystallography and electron spin resonance (ESR) can unfold their power in defined model systems but underlie the persistent limitation that lipid/protein interactions cannot be probed in complex native, living systems—and if they can, they yield only indirect information and interpretation of data is difficult. Even state of the art imaging techniques lack the spatial and temporal resolution to probe transient interactions *in situ*. Molecular dynamics simulations have identified a tightly associated ring of annular lipids around the potassium channel Kv1.2 that even extended several nanometers from the protein surface,^20^ but considerably smaller effects have been detected for other proteins.^20,21^ Ultimately, results of MD simulations depend on the choice of force fields and are limited by the accessible time scales and complexities of the system.

Here, we employed a previously developed protein micropatterning assay^22,23^ to probe the nanoenvironment of different transmembrane proteins in the live cell plasma membrane (Figure 1A). The assay exploits the fact that immobilized obstacles reduce the diffusivity of tracer molecules in a characteristic manner, enabling inference of obstacle size. Importantly, it does not require high-resolution tracking of individual tracer molecules but instead relies on measuring average diffusion constants. The precision of the assay critically depends on robust determination of the fold reduction in tracer diffusivity caused by immobilized obstacles, which can only be achieved by performing measurements with and without immobilized obstacles in the very same cell. To this end, a protein of interest (POI) is enriched and immobilized in specific areas directly in the plasma membrane of a living cell (“ON” areas, Figure 1B), leaving other areas depleted of the POI (“OFF” areas). Lipid tracer molecules diffusing in ON areas experience the immobilized POI molecules as obstacles to their random walk, which lowers their diffusional mobility as a function of the POI surface density and size.^22,24,25^ Additionally, the specific, coimmobilized lipid nanoenvironment surrounding the POI can influence tracer diffusion in three ways (Figure 1B): (i) the tracer is not affected at all; (ii) the tracer is excluded from a shell of tightly associated lipids; and (iii) the tracer can enter the nanoenvironment, where it is slowed down or even transiently coimmobilized with the POI. In cases (ii) and (iii), the experiment will yield an apparent POI size that is larger than the steric size of the POI.

**Figure 1:**
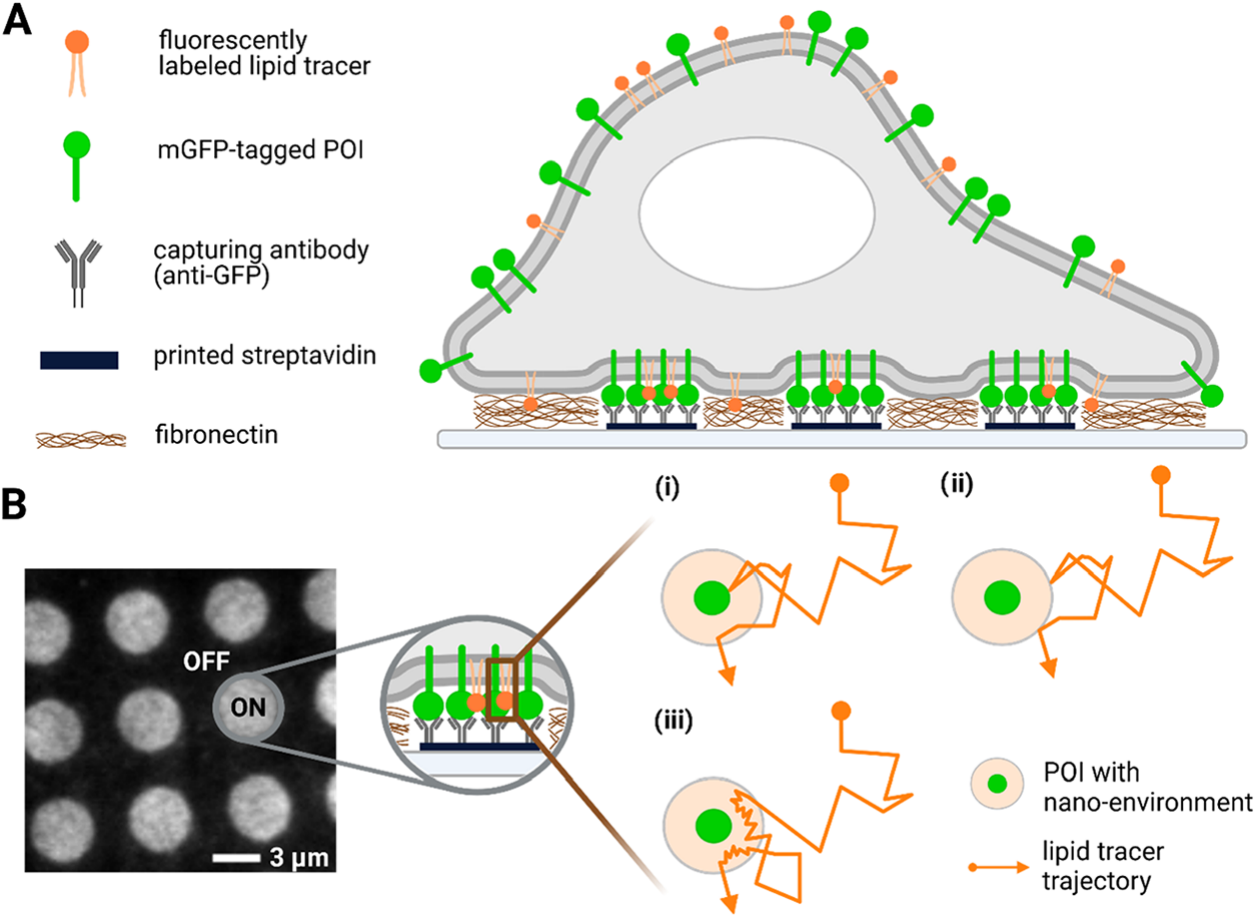
Lipid tracer diffusion in micropatterned plasma membranes. (A) mGFP-tagged plasma membrane POIs are enriched and immobilized according to GFP antibody patterns in ON areas, leaving OFF areas depleted. (B) Region of interest in a total internal reflection fluorescence microscopy (TIRFM) image of a cell grown on micropatterns. Immobilized POI molecules pose steric obstacles to diffusion, thus decreasing average tracer mobility in ON areas compared to OFF areas (i). Tracer mobility will be further decreased if the nanoenvironment surrounding the POI is impenetrable to the tracer (ii) or slows down the tracer diffusion upon entry (iii), thus yielding a larger apparent in-plane radius of the obstacle.

Using the decrease of lipid tracer mobility in ON vs OFF areas as a readout parameter, we found that experimentally determined POI sizes were generally in good agreement with structural data. By relating experimental to simulated data, we were able to define boundaries for possible nanoenvironment properties, allowing us to rule out even a single layer of tightly associated boundary lipids for three out of the four chosen POIs.

## Results

### Tracer Diffusion in Micropatterned Cells

Microstructured glass coverslips featuring regular anti-GFP antibody patterns of 3 μm-sized dots were created by soft lithography as described previously.^23,26^ HeLa cells expressing mGFP-tagged proteins of interest exhibited specific enrichment and immobilization of the POIs within the live cell plasma membrane according to the antibody pattern (Figure 1A). The density of immobilized POIs per μm^2^, ρ_POI_, within ON areas was adjusted via the GFP antibody density and the POI expression level. We chose representatives of different transmembrane protein classes for our experiments: (i) the β2-adrenergic receptor (β2-AR), a G-protein coupled receptor (GPCR) with 7 transmembrane helices and an N-terminal mGFP; (ii) the putatively hexameric calcium channel Orai1,^27^ consisting of 4 transmembrane helices, featuring an mGFP in an extended loop 3; (iii) a single-pass transmembrane protein construct based on influenza virus hemagglutinin (HA-WT), and (iv) the palmitoylation-deficient mutant thereof (HA-Δpalm). The latter two proteins contained the transmembrane and cytoplasmic domains of HA, as well as a truncated exoplasmic moiety, to which mGFP was fused; the wildtype protein featured three palmitoylation sites proximal to the transmembrane region. As tracer lipid, we chose a fluorophore-tagged sphingomyelin (SM-Atto594),^28^ where a short nonaethylene glycol linker reduces partitioning of the fluorophore to the membrane interface region and prevents flipping of the lipid to the intracellular leaflet of the bilayer. We doped the plasma membrane with SM-Atto594 at densities that allowed us to distinguish well separated single molecule signals and used total internal reflection fluorescence microscopy (TIRFM) to determine its diffusional mobility. For analysis, single molecule trajectories were classified as “ON” and “OFF” using a selection mask derived from the POI pattern image recorded in the GFP channel (Figure 1B and Supporting Figure S2). Trajectories were pooled separately for each cell’s ON and OFF areas, and diffusion coefficients *D*_ON_ and *D*_OFF_ were determined from the mean square displacements (MSD) by fitting the first two lag-time points (Figure 2A and Supporting Figure S1; for details see Methods section). Typically, tracer mobility was well described by Brownian motion at time lags up to ∼50–100 ms in both ON and OFF areas; at longer time lags, application of the selection mask introduces an artificial confinement, resulting in apparent subdiffusion behavior (Supporting Figure S3). We confirmed that micropatterning or application of the selection mask *per se* did not have unintended effects on the measured diffusion coefficients (Supporting Table S1 and Figure S3).

**Figure 2:**
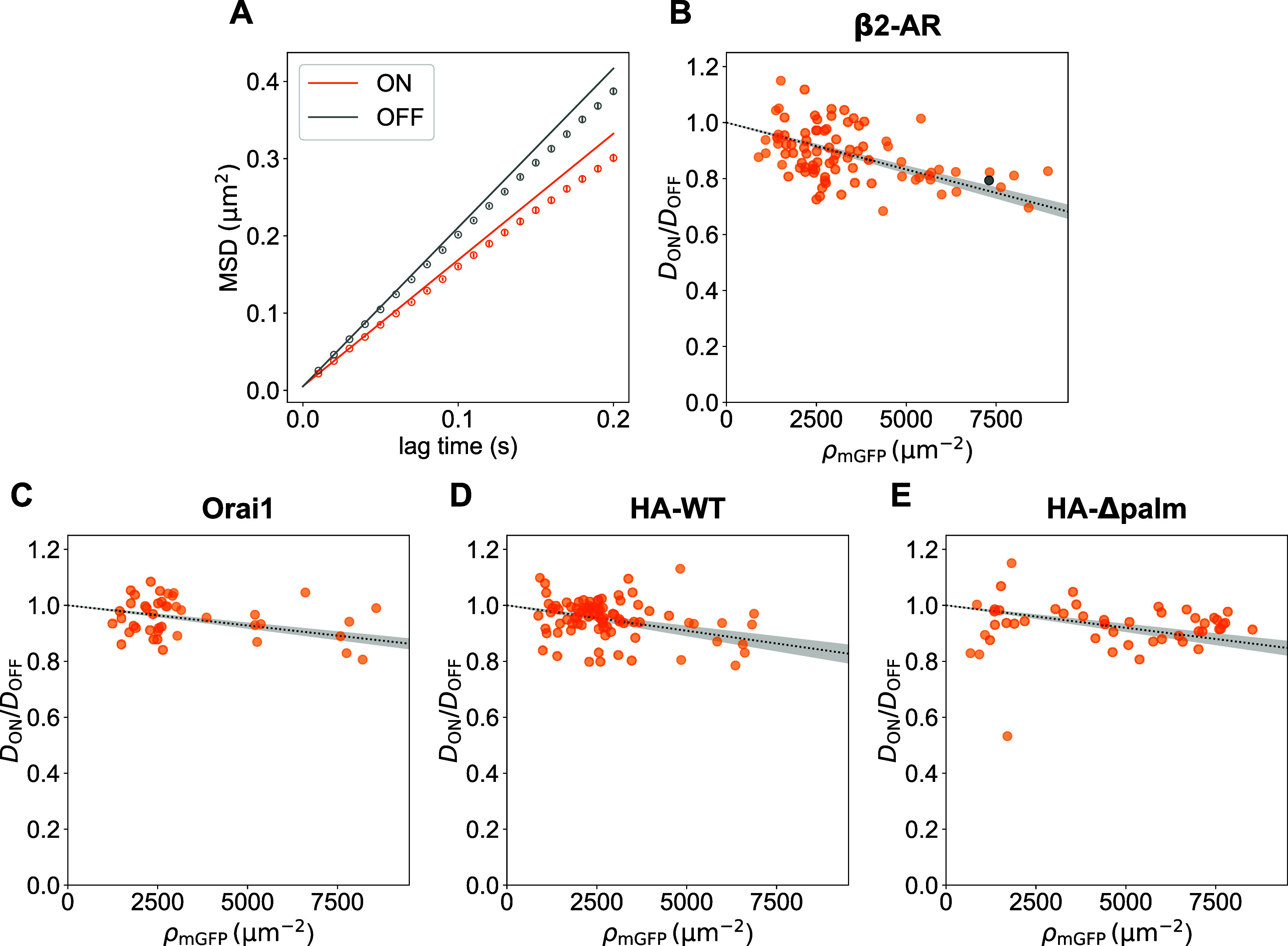
Tracer mobility decreases with increasing density of immobilized protein. (A) Mean square displacement (MSD) of the tracer molecule SM-Atto594 as a function of lag-time *t*_lag_ recorded in ON (orange) and OFF (gray) areas for a representative cell expressing β2-AR-mGFP. Relative diffusion coefficients *D*_ON_/*D*_OFF_ were determined for each individual cell, plotted versus the mGFP density measured in ON areas, ρ_mGFP_, and fitted with eq 1. Data are shown for cells expressing mGFP-tagged β2-AR (*n* = 88 cells) (B), Orai1 (*n* = 43 cells) (C), HA-WT (*n* = 85 cells) (D) and HA-Δpalm (*n* = 48 cells) (E). Fits are shown as black dotted lines with areas shaded in gray indicating fit errors (see Methods section). The gray dot in (B) indicates the cell shown in (A).

Next, we determined the surface density of immobilized mGFP-tagged POI, ρ_mGFP_, by relating the bulk ON brightness to the brightness of single mGFP molecules (see Methods section) and plotted the relative mobility *D*_ON_/*D*_OFF_ as a function of ρ_mGFP_ (Figure 2B–E). As expected and observed previously,^22,23^ *D*_ON_/*D*_OFF_ decreased with increasing ρ_mGFP_ for all patterned proteins.

### Determining Protein Size from Tracer Diffusion

Immobilized proteins act as steric obstacles to the diffusion of tracer molecules. In the absence of other interactions, tracer diffusion relates to the density of randomly immobilized circular obstacles ρ and their size via

1 $$\frac{D_{\mathrm{ON}}}{D_{\mathrm{OFF}}}=\left(1-\frac{{1-e}^{-\rho R^{2}\pi }}{C_{P}}\right)$$

with *C*_P_ being the percolation threshold, i.e., the obstacle density at which long-range conducting paths for tracer diffusion disappear and the diffusion coefficient approaches zero.^24,25^ In this model, the radius of tracer and obstacle are simplified to a combined radius *R*.^25^ Modeling obstacles as overlapping discs, the area coverage of obstacles at the percolation threshold is independent of the obstacle size and given by *C*_P_ ≈ 0.676.^25^

We have previously performed Monte Carlo simulations of tracers diffusing through regions of immobilized obstacles and coimmobilized nanoenvironments and evaluated the effect of different nanoenvironment sizes and diffusivities.^24^ From this, we developed an empirical model that describes the tracer mobility ratio *D*_ON_/*D*_OFF_ in the presence of inert steric obstacles with coimmobilized nanoenvironments. Our simulations had revealed that the three scenarios shown in Figure 1B affect *D*_ON_/*D*_OFF_ in a qualitatively similar manner at our experimental time scales and obstacle densities; the presence of lipid nanoenvironments would only be detectable as an increased in-plane POI radius. We hence fitted the experimental data with a model of inert circular obstacles (eq 1). For this, the measured density ρ_GFP_ was corrected to account for incomplete GFP maturation^29^ and the oligomeric state of the protein (see Methods section). Applying eq 1 yielded the experimentally determined apparent radius *R*_app_, which also includes the lipid tracer size *R*_lipid_. Based on the area per lipid of 0.625 nm^2^ determined for C18-SM from SAXS/SANS data^30^ and factoring in the bulky triazole moiety^31^ in the headgroup region, we estimated a tracer radius of *R*_lipid_ = 0.49 ± 0.05 nm for SM-Atto594 ^28^ (for details see Methods section). Subtracting the tracer size *R*_lipid_ from *R*_app_ yields the apparent in-plane protein radius *R*_POI,app_ = *R*_app_ – *R*_lipid_, which incorporates the steric size of the protein plus potential contributions from its membrane nanoenvironment.

### Comparison of Apparent Protein Sizes with Protein Structures

To evaluate our experimentally determined *R*_POI,app_ data we first considered available protein structural information to obtain a reasonable estimate for the steric size of the protein obstacle the tracer would encounter. From a recent NMR structure of β2-AR^32^ we extracted the positions of all atoms in the outer membrane leaflet and determined the cross-sectional area of a convex hull around their z-projection. From this, we derived the in-plane radius of a hypothetical circular obstacle of identical area, *R*_POI_ *=* 1.94 nm. β2-AR may form functional homodimers at the plasma membrane;^33^ based on a crystal structure that indicated a dimer bridged by cholesterol^34^ we determined a dimer radius *R*_POI_ = 3.13 nm. The aspect ratio of the cross-sectional area in this case was ∼2:1, which is still in a regime where the influence of obstacle shape on *C*_P_ is minor^35–37^ and can be neglected for our purposes (Supporting Figure S4).

While there is no crystal structure available for human Orai1, an ortholog from *Drosophila melanogaster* has been crystallized as a hexamer.^27^ Using the same approach as for β2-AR we obtained a hexamer radius *R*_POI_ = 3.16 nm. For HA-WT and HA-Δpalm, we calculated the protein radius based on the 3D structure of the transmembrane helix predicted by AlphaFold2 ^38^ with the orientation in the membrane calculated via PPM 3.0.^39^

Figure 3 shows a comparison of our experimentally derived apparent protein radii with the corresponding radii extracted from structural data. For β2-AR, the measured radius was in good agreement with the radius estimated from structural data, irrespective whether a monomeric or dimeric structure was assumed (Table 1 and Supporting Figure S5). The experimental data for Orai1 yielded a slightly larger size than predicted (3.54 ± 0.28 vs 3.16 nm). The measured sizes for HA-WT and HA-Δpalm were comparable (1.37 ± 0.09 and 1.25 ± 0.11 nm, respectively), but also slightly larger than expected from structure predictions (1.06 nm). In summary, experimentally determined POI radii were generally similar to those extracted from structural data.

**Figure 3:**
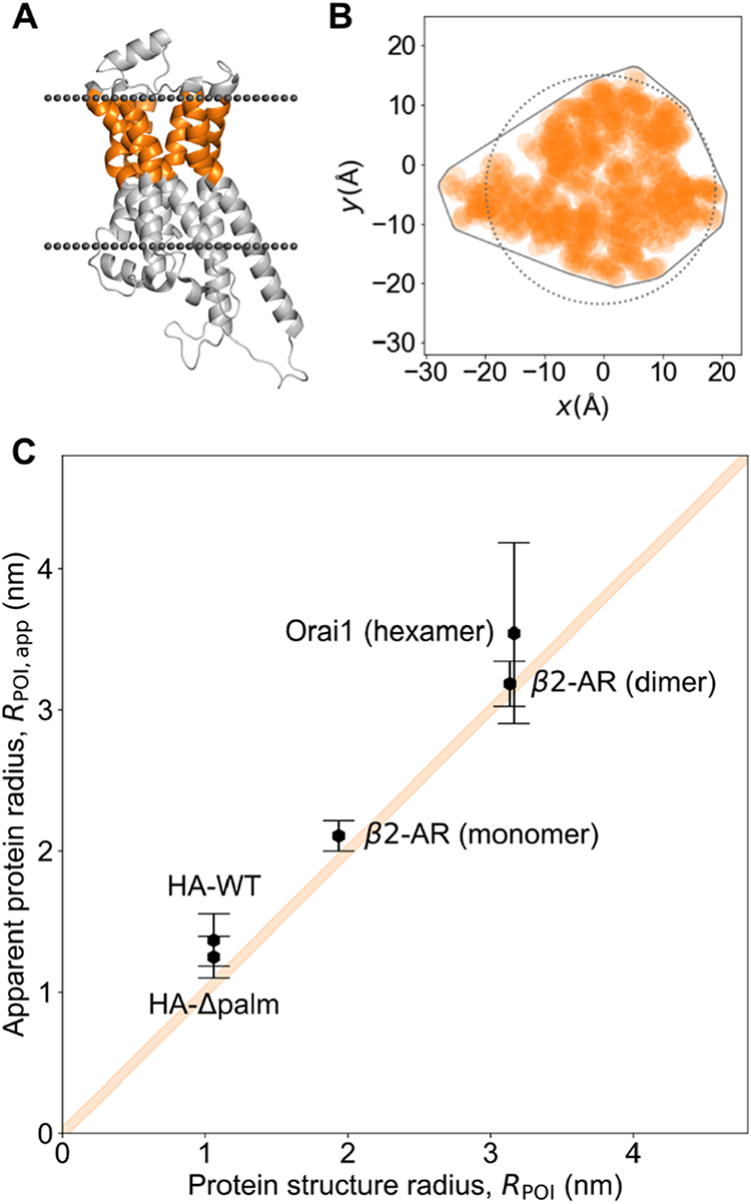
Comparison of experimentally determined protein radii with protein structures. (A) NMR structure of β2-AR.^32^ For determining the protein size *R*_POI_, the protein moiety residing in the extracellular leaflet (indicated in orange) was considered and its atoms were plotted as a z-projection (B). A convex hull was applied and from its cross-sectional area the radius of a hypothetical circular obstacle *R*_POI_ of equal area was derived. (C) Experimentally determined protein sizes *R*_POI,app_ are plotted versus *R*_POI_. The orange line indicates equal size taking into account the size of the lipid tracer with *R*_lipid_ = 0.49 ± 0.05 nm. *Y*-axis error bars show the error of the fit (see Methods section) using eq 1.

**Table 1:** Experimentally Determined Apparent Protein Radii *R*_POI,app_ in Comparison with Radii Extracted from Structural Data *R*_POI_

	oligomeric state	*R*_POI_ (nm)	*R*_POI,app_ (nm)^a^
β2-AR	monomer	1.94	2.11 ± 0.06
	dimer	3.13	3.19 ± 0.09
Orai1	hexamer	3.16	3.54 ± 0.28
HA	monomer	1.06	1.37 ± 0.09
HA-Δpalm	monomer	1.06	1.25 ± 0.11

a The error of the lipid radius (±0.05 nm) is not included.

### Possible Characteristics of Protein-Associated Lipid Nanoenvironments

Up to now we have considered POIs as obstacles impermeable to the lipid tracer. However, we may well expect POI-associated lipid nanoenvironments that allow the entry of the tracer and only reduce its diffusivity therein (Scenario iii in Figure 1). We were thus interested, how the presence of such putative nanoenvironments with reduced diffusivity (NERDs) would be detected in our experiments. To examine this, we made use of our previously developed empirical model that describes the tracer mobility as a function of the density of inert steric obstacles with coimmobilized NERDs^24^ (eq 3). For our model we assume circular POIs of radius *R*_POI_ surrounded by a ring-shaped lipid nanoenvironment with a width *d*_NERD_. Within the NERD, tracer mobility decreases by a factor of *f*_NERD_ relative to the bulk membrane area (Figure 4A).

**Figure 4:**
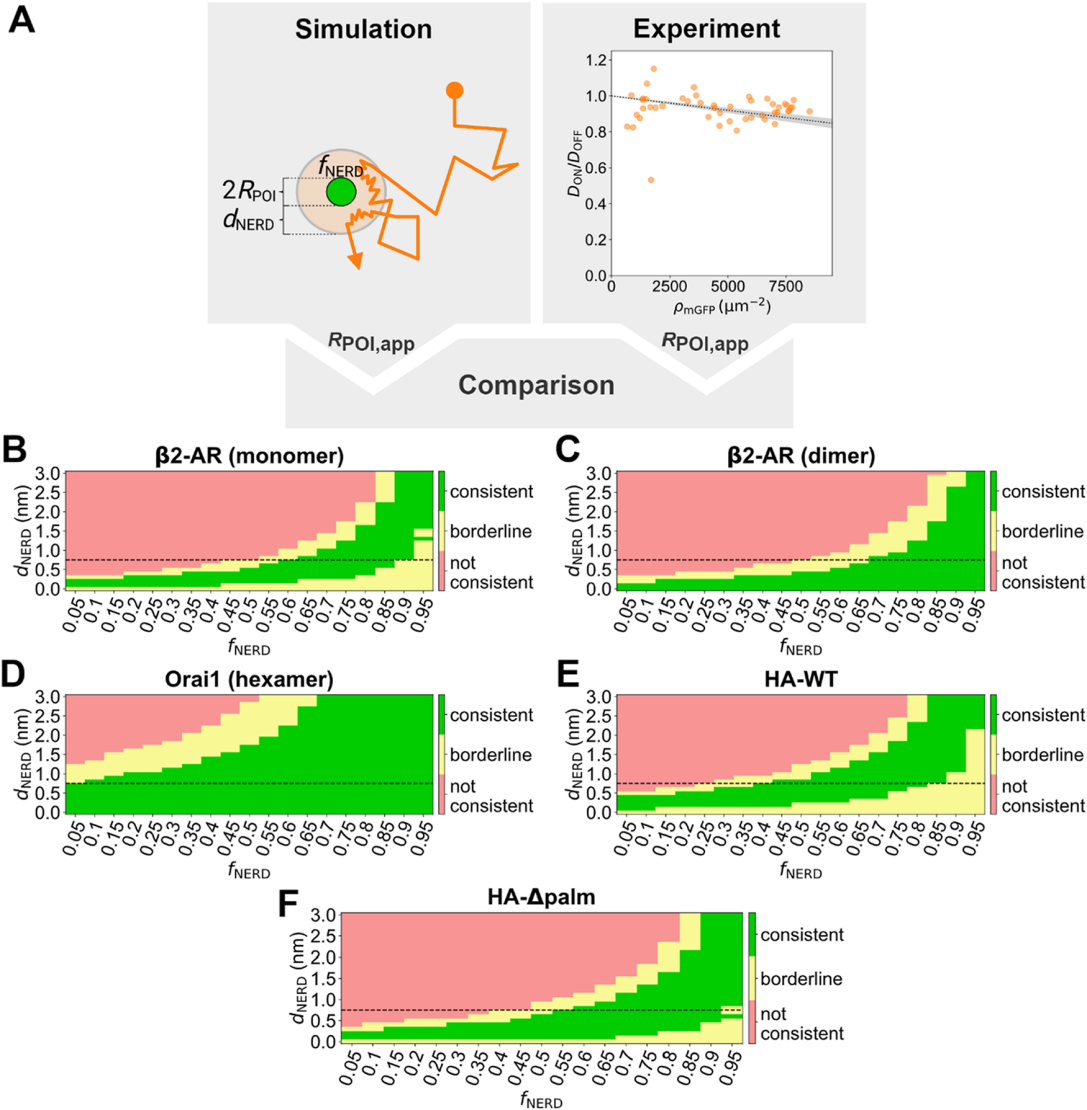
Possible characteristics of POI-associated nanoenvironments. (A) Workflow of determining NERD characteristics that are consistent with experimental data. Immobilized proteins with *R*_POI_ are assumed to be surrounded by NERDs with a width *d*_NERD_, where tracer mobility is reduced by a factor *f*_NERD_ compared to the bulk membrane (OFF) area. Comparison of simulated to experimental data allows for identifying NERD characteristics. (B–F) *R*_POI,app_ values are calculated for 133 different (*d*_NERD_, *f*_NERD_) combinations via eq 3, compared with the experimentally determined *R*_POI,app_ values and categorized as consistent (green; mean of experimental data is within the error of modeled data), borderline (yellow; errors overlap), and not consistent (red; errors do not overlap) (see also Supporting Figure S7). The dashed line indicates a d_NERD_ of 0.7 nm, corresponding to a single layer of lipids.

Evidently, there are combinations of *d*_NERD_ and *f*_NERD_ that give rise to identical dependencies of *D*_ON_ */D*_OFF_ on ρ_POI_. For example, large values of *d*_NERD_ combined with a minor mobility decrease *f*_NERD_ would affect the data in a similar way as small values of *d*_NERD_ combined with a large mobility decrease. We were hence interested, which combinations of *d*_NERD_ and *f*_NERD_ would be consistent with our experimental data.

To this end, we explored 133 different combinations of *d*_NERD_ and *f*_NERD_. For each combination, we predicted the dependence of *D*_ON_/*D*_OFF_ on the POI density ρ_POI_, and analyzed these computer-generated data sets in an identical way as our experimental data sets (for details see Methods section): we fitted each data set to eq 1 and accounted for the lipid size, yielding values *R*_POI,app_ for the different NERD sizes and diffusivities (Supporting Figure S7). Next, we compared experimentally determined *R*_POI_,_app_ values with the modeled ones to identify NERD characteristics that are consistent with our data (indicated in green in Figure 4B–F) and those that we can rule out (indicated in red). For β2-AR, HA-WT and HA-Δpalm, our data would be consistent with NERDs with *d*_NERD_ = 0.7 nm (a single layer of lipids) and moderately reduced mobility compared to the bulk membrane area (*f*_NERD_ ≥ 0.5), as would NERDs with *d*_NERD_ ≤ 1.4 nm if the mobility was reduced by less than 30%. Our data allow us to exclude the presence of even a single layer of lipids surrounding the protein core with diffusivities reduced by 90% (*f*_NERD_ ≤ 0.1) (Figure 4). In the case of Orai1, the parameter space is larger; NERD sizes up to *d*_NERD_ = 2.1 nm (∼3 layers of lipids) and 50% reduced diffusivity would be consistent with our data, as would be up to 2 layers of lipids with 85% reduced diffusivity (Figure 4D).

## Discussion

It is well-established that transmembrane proteins exert an influence over the dynamics, conformation, and organization of lipids in their immediate vicinity. Apart from a moderate number of well-characterized specific lipid interactions, however, the extent of this influence is largely unknown. Among the discussed concepts are enrichment of specific lipid species around transmembrane proteins that mediate function,^1,6,17^ as well as tightly associated lipid shells that codiffuse with a protein.^20,21^ Although often observed in simulations, experimental evidence validating these concepts is scarce and challenging to obtain. By making use of a protein micropatterning approach, we addressed this gap, asking a simple question directed at this fundamental aspect of plasma membrane organization: How does a transmembrane protein affect the fluidity of its immediate lipid nanoenvironment?

For our study, we chose proteins from four different classes, for which various more or less specific lipid interactions had been reported, with the rationale that these differences might also impact their idiosyncratic lipid nanoenvironment.^1,6,7,17^ The interaction of β2-AR with cholesterol is well-documented: cholesterol has been cocrystallized,^40^ proposed to change the protein’s structural properties^41^ and three specific binding sites have been identified for allosteric modulation of its activity.^42^ For the truncated HA variant used in this study, a palmitoylation-dependent coclustering was found with GPI-CFP in a fluorescence lifetime microscopy - Förster resonance energy transfer (FLIM-FRET) study, which was interpreted as partitioning into membrane rafts.^43^ Palmitoylation increases a protein’s hydrophobicity due to the attachment of saturated acyl chains and palmitoylated proteins have often been found to partition to ordered phases in phase-separated model systems and detergent-resistant membranes (DRMs).^44^ Orai1 can be palmitoylated^45^ and bind cholesterol,^46^ both of which have been shown to modulate its function.

In our assay, we used the decrease of tracer mobility in the presence of a defined density of immobilized POIs as readout parameter for determining the size of the protein obstacle experienced by the tracer.^22,25,47^ In general, protein sizes in the live cell plasma membrane were in good agreement with those deduced from available structural data. Specifically, for none of the examined proteins did we detect a tightly associated shell of lipids impenetrable to the SM-Atto594 tracer, which would have been evidenced by a radius increased by ∼0.7 nm.

Employing our previously developed empirical model,^24^ we systematically mapped the parameter space of lipid nanoenvironments potentially associated with the proteins, focusing on their size and effect on diffusivity. While we cannot provide a definitive characterization of the nanoenvironments surrounding the studied proteins, our analysis enables us to establish boundaries for potential scenarios. If we assumed only the first layer of lipids in direct contact with the protein being affected (i.e., *d*_NERD_ = 0.7 nm), the largest possible effect supported by our data would be a reduction of lipid mobility within this lipid shell (*f*_NERD_ in Figure 4) to 50, 5, 25 and 40% for β2-AR, Orai1, HA-WT and HA-Δpalm, respectively. Let us compare our results with computer simulations of protein diffusion in lipid bilayers. Niemelä et al. used atomistic molecular dynamics simulations to study the influence of the voltage-gated ion channel Kv1.2 on its lipid nanoenvironment.^20^ In that study, the authors described a 15-fold reduction of lipid mobility in immediate contact with the protein surface and, on top of this, a significant, albeit smaller impact on adjacent lipid layers, with approximately 50–100 lipids (equivalent to 2–3 layers) moving together with the ion channel. In our experimental settings, this scenario would manifest as a coimmobilization of lipids detected as a nanoenvironment with at least *d*_NERD_ = 0.7 nm (a single lipid layer) and a reduction in mobility with *f*_NERD_ < 0.06. This effect is considerably larger than what was typically observed in our experiments; only data obtained for Orai1 would be consistent.

It does seem plausible that the extent of mobility reduction of lipids in direct contact with the protein depends on the specific characteristics of the transmembrane domain. Proteins with a highly corrugated surface, such as the Kv1.2 channel,^20^ may promote tighter association of lipids. Indeed, MD simulations of the 12-transmembrane helix protein LacY,^20^ a WALP23 dimer^20^ and the single-spanning transferrin receptor^21^ revealed a qualitatively similar, but somewhat smaller effect on lipid mobility in close vicinity to the protein.

Orai1 assumes a distinct role in our study. Due to its hexameric state, we could not achieve as high surface densities of patterned protein as for the other POIs, resulting in larger errors for the apparent radius. This contributes to the larger NERD parameter space observed for Orai1—our experimental data would be consistent with the presence of up to two layers of tightly associated lipids (where the mobility is reduced to 15%). It does, however, not explain the difference between sizes extracted from experiment and crystal structure (*R* = 3.54 nm and *R* = 3.16 nm, respectively). Note that for estimating the size from the crystal structure only protein and lipid moieties that reside within the exoplasmic leaflet were considered. This simplification appears generally justified since the extracellular parts of all POIs are smaller or of equal size as compared to the transmembrane regions (Supporting Figure S5). The contribution of mGFP in the fusion proteins may, however, be relevant, particularly in the case of Orai1. Orai1 hexamers can be attached to the patterned antibodies via up to six mGFP molecules, resulting in a size of the extracellular membrane-proximal region of *R* = 4.9 nm (assuming a perpendicular orientation of mGFP; for details see Supporting Figure S6), which is considerably larger than the transmembrane region (*R* = 3.16 nm). We thus cannot rule out that steric hindrance and/or interactions of the fluorophore on the lipid tracer and the Orai1-conjugated mGFPs contribute to the experimentally determined size, and thus lead to an overestimation of the extent of lipid association to Orai1. In principle, this also applies to β2-AR, HA-WT and HA-Δpalm, although possible contributions from extracellular moieties and/or mGFP are smaller in these cases (Supporting Figure S6).

Since our lipid tracer exclusively probes the exoplasmic leaflet, NERDs only present in the cytoplasmic leaflet would not be detected in our assay. Particularly, this could pertain to β2-AR, for which allosteric modulation by anionic phospholipids has been reported.^48^ Further, an increased size of HA-WT due to palmitoylation and/or a more viscous nanoenvironment around HA-WT that does not extend to the exoplasmic leaflet, would not register.

In the literature, lipids of a (putative) nanoenvironment are often referred to as “different from bulk lipids”. Considering the abundance of proteins in the plasma membrane (50% of its mass^49^ and ∼30,000 proteins per μm^2^,^50^ ∼15% of its total area is occupied by protein^49^), the existence of such “bulk” or “free” lipids has been questioned.^51^ Let us assume that all lipids are affected in their diffusion by proteins and the plasma membrane consists only of nanoenvironments—how would this affect our results? Immobile obstacles have a vastly higher impact on tracer diffusion than a mobile obstacle.^52^ By micropatterning a protein of interest, it is thus singled out via immobilization, and along with it, its specific nanoenvironment. With the majority of plasma membrane proteins being mobile, a lipid tracer in OFF regions would move between different (mobile and immobile) nanoenvironments with *D*_OFF_. Within ON regions, it would also encounter a high density of the immobilized POI (and their nanoenvironments), with a dominant effect on tracer diffusion. Therefore, even if there are no bulk lipids in the plasma membrane, boundary lipids associated with the immobilized POI would be detected in our assay.

## Conclusion

In conclusion, our data indicate that tight association of annular lipids around transmembrane proteins is not a generalizable principle of plasma membrane organization. Note that this finding pertains to resting, unstimulated cells; small changes in the membrane energy landscape may be sufficient to overcome the entropic penalty of lipid–protein association. A lipid nanoenvironment around a protein may be nucleated or stabilized, for instance, by protein clustering induced via receptor-mediated signaling.^19^ This could happen intertwined with or originating from events at the intracellular leaflet, via charge interactions between protein and lipids,^53–55^ membrane-proximal liquid–liquid phase separation in the cytosol^56–58^ and/or coupling to the cortical actin cytoskeleton.^59,60^

## Methods

### Constructs

POI sequences were cloned into expression vectors and fused with extracellular monomeric enhanced GFP (mGFP). For the Orai1 construct, we generated an Orai1-Orai3 chimera, which contained Orai1 (res. M1-A578, UniProt Q96D31) with the loop 3 (res. F199–I242) replaced by that of Orai3 (res. F174–I251, UniProt Q9BRQ5) using Gibson Assembly. The Orai1 loop3-Orai3 chimera was assembled in a YFP-C2 vector and then cloned into a modified N1 vector via *Eco*RI and SacII. A *Kpn*I site was introduced within loop3 using the Stratagene Site-Directed Mutagenesis system. In a next step, mGFP was amplified by PCR and cloned via *Kpn*I into the loop3 of the Orai1 loop 3-Orai3 chimera, yielding the Orai1 loop 3-Orai3-mGFP chimera. For transient transfections, the Orai1 construct was cloned into a pcDNA3 vector (Invitrogen). The β2-AR sequence (res. G2-L413, natural variant bearing the mutations G16R and E27Q, UniProt P07550) fused with an N-terminal mGFP cloned into the pcDNA3 vector was a gift from M. Ulbrich, University of Freiburg. To ensure correct membrane targeting, the secretion signal from serotonin receptor 3A (res. M1-R25, with A19G exchange, UniProt Q8K1F4) was leading the protein sequence. The sequence of HA-WT consisted of an ER translocation sequence (MELFWSIVFTVLLSFSCRGSDWESLQSTVPR) followed by mGFP and a sequence of viral hemagglutinin (res. A492-I563, UniProt Q84027) and was cloned from HA-mEos3.2 ^61^ (gift from S. Veatch, University of Michigan). Cysteines at palmitoylation sites were mutated to serines (C551S, C559S, C562S) to create HA-Δpalm. For the generation of stably expressing cells using an inducible piggyBac system,^62^ HA-WT and HA- Δpalm constructs were cloned into PB-T-PAF. This vector was used together with PB-RB (both plasmids were a gift from Prof. James M. Rini, University of Toronto) and PBase (obtained from Wellcome Trust Sanger Institute, Hinxton, United Kingdom).

### Cell Culture

HeLa cells (DSMZ. no. ACC57, Leibniz Institute DSMZ, Berlin, Germany) were cultured in Dulbecco’s Modified Eagle’s Medium (DMEM; Sigma-Aldrich, D6429 and Gibco, 11995) supplemented with 10% fetal bovine serum (FBS; Sigma-Aldrich, F75524) and maintained in a humidified atmosphere with 5% CO_2_ at 37 °C. To investigate Orai1and β2-AR, we transiently transfected the cells using TurboFect (Thermo Scientific, R0531). Cells were used in experiments within 24–48 h after transfection. For HA-WT and HA-Δpalm, an inducible piggyBac expression system was additionally used. HeLa cells were transfected using TurboFect with the piggyBac vectors (PBase, PB-RB, and PB-T-PAF encoding respective GFP-tagged proteins in a 1:1:8 ratio). Selection antibiotics (0.5 μg/mL puromycin, 1.2 μg/mL blasticidine) were added 48 h after transfection. As soon as the cells recovered and reached optimal viability (14–21 days), cell lines were expanded, kept in the culture, or frozen at −80 °C as a backup. Protein expression was induced by addition of 500 ng/mL of doxycycline 16–48 h before the experiments. For micropatterning experiments, cells were grown to 60–80% confluency and harvested by treatment with accutase (Sigma-Aldrich).

### Total Internal Reflection Fluorescence Microscopy (TIRFM)

TIRFM measurements were performed using a home-built microcopy setup based on an inverted Axiovert 200 microscope (ZEISS) equipped with an α Plan-Apochromat objective (100*×*/1.46 oil DIC (UV) VIS-IR). For excitation of fluorophores, lasers at wavelengths 488 nm (iBeam-Smart-CD) and 561 nm (Coherent Obis Galaxy Laser 1275608) were used. To achieve TIR illumination the excitation beam was shifted away from the optical axis by a mirror on an adjustable piezo-controlled table. A dichroic mirror (zt488/561rpc, Chroma F53–495 AHF) was used in combination with a dual-band emission filter (Chroma, F57–019 AHF) to selectively reflect excitation light and transmit emitted light, which was then focused by a 1*×* tube lens onto an Andor iXon Ultra EM-CCD camera with 16 μm pixel size (operated at −60 °C).

### Micropattern Preparation

Microstructured surfaces were prepared as described.^26^ Briefly, PDMS stamps featuring circular pillars of 3 μm diameter and 6 μm center-to-center distance were cleaned with absolute ethanol and ddH_2_O, then incubated with 50 μg/mL streptavidin (Sigma-Aldrich, S4762) for 15 min at room temperature. Excess streptavidin was rinsed off with ddH_2_O and stamps were dried thoroughly under a stream of nitrogen. Dried stamps were placed onto epoxy-coated glass coverslips (Schott Nexterion CoverslipE (0.175 ± 0.02 mm)). After 1 h of incubation at room temperature stamps were removed, Secure-Seal hybridization chambers (Grace Bio-Laboratories) were placed on top of the structures and 50 μg/mL fibronectin (Sigma-Aldrich, F1141) in PBS was added. After 30 min at room temperature or overnight at 4 °C, 10 μg/mL biotinylated monoclonal 9F9.F9 GFP antibody (NOVUS Biologicals) in PBS with 1% BSA was added. After 15 min, samples were rinsed thoroughly with PBS.

### Labeling of Cells with Tracer Lipid

For labeling with the tracer lipid SM-Atto594,^28^ detached HeLa cells expressing the mGFP-tagged protein of interest were incubated with 5 nM of the lipid in media at 37 °C for 15 min. The tracer lipid was removed by repeated centrifugation and washing steps, and cells were seeded onto the micropatterned coverslips. Cells were left to attach for 30 min at 37 °C. For all measurements buffers were exchanged to HBSS (Sigma-Aldrich H8264) containing 2% FBS.

### Lipid Tracer Diffusion

The movement of lipid tracers within cells was recorded for 500 frames with an illumination time of 3 ms and a delay of 7 ms using a 561 nm laser at 0.25–0.3 kW/(cm)^2^ in TIR configuration. Typically, 8–10 movies were recorded per cell, yielding 100,000–200,000 single-molecule localizations and ∼2000–3000 trajectories (Supporting Table S2). Single tracer molecules were localized using the open-source Python package sdt-Python (doi: 10.5281/zenodo.6802801), with an average localization precision of 40 nm. Here, we opted to use the implemented daostrom algorithm.^63^ Localized molecules were tracked using trackpy, an open-source Python package. Generated trajectories were divided into two groups, ON and OFF, using selection masks based on the recorded mGFP patterns of the respective cell. Mean square displacements (MSD) within ON and OFF areas of cells were plotted against the time lag (*t*_lag_).

Considering Brownian motion and correcting for illumination time, MSDs relate to *t*_lag_ according to

2 $$\mathrm{MSD}\left(t_{\mathrm{lag}}\right)=4D\left(t_{\mathrm{lag}}-\frac{1}{3}t_{\mathrm{ill}}\right)+{4\sigma }^{2}$$

with σ denoting the localization precision and *D* denoting the diffusion constant.

MSD vs *t*_lag_ plots slightly deviated from Brownian motion for high values of *t*_lag_ as a consequence of application of the selection mask (see Supporting Figure S3). Hence, MSD vs *t*_lag_ plots were fitted with eq 3 using the first two data points only to determine diffusion constants. Errors of diffusion constants were determined by bootstrapping.

### Determination of Patterned Protein Density

The density of immobilized POI per μm^2^ within ON areas, ρ_POI_, was adjusted via the GFP antibody density and the POI expression level. It was determined by relating the mean bulk brightness of the POI-mGFP-enriched ON areas with the brightness of single mGFP molecules as described previously.^22^ For the former, the GFP signal of a micropatterned cell was recorded by illuminating the sample for 3 ms with a 488 nm laser at 0.02–0.05 kW/(cm)^2^ using TIR excitation. To determine the brightness of a single GFP molecule, POI-mGFP expressing cells were seeded onto a fibronectin-coated coverslip. A rectangular region of interest was photobleached using a strong laser pulse to achieve single molecule densities. Well-separated mGFP signals were then recorded in TIRF configuration for a sequence of 100 frames with an illumination time of 20 ms and a delay of 30 ms between consecutive frames. Images of patterned cells as well as single-molecule images were flat-field corrected. Single molecules were localized as described above. Single mGFP brightness was calculated as the median of integrated brightness values of all molecules typically starting from frame 30–50 to avoid the detection of multimeric POI-mGFP species. The mean pixel intensity values of OFF regions were subtracted from the mean pixel intensity values of ON regions to determine the intensity contribution of immobilized POI-mGFP species. The resulting mean pixel intensity of immobilized POI-mGFP was divided by the single molecule brightness (adjusted for the illumination time) to yield the surface density ρ_GFP_, which was then corrected to account for incomplete maturation of the mGFP chromophore with 20% dark mGFP.^29^ Taking the oligomeric state *n* of the POI into account, we calculated the density of POI oligomers ρ_POI_ = ρ_mGFP_ × 1.2/*n*.

### Fitting Experimental Data to Determine the Apparent POI Radius *R*_POI,app_

Experimental data corrected for incomplete mGFP maturation was fitted with eq 1, yielding *R*_app_, which also includes the lipid tracer size *R*_lipid_. Subtracting the tracer size *R*_lipid_ from *R*_app_ yields the apparent in-plane protein radius *R*_POI,app_ = *R*_app_ – *R*_lipid_, which incorporates the steric size of the protein plus potential contributions from its membrane nanoenvironment. To estimate fit errors, we simulated 1000 data sets with eq 1 corresponding to the determined value for *R*_app_ taking into account inherent characteristics of our experimental data (distribution of measured densities, measurement errors in density and diffusion as well as the overall number of recorded cells). Each of these data sets was then fitted using eq 1. The standard deviation of all determined values for *R*_app_ yields its variability and is used as an estimate for the fit error.

Since β2-AR may form monomers or dimers in the plasma membrane, we chose to reanalyze our data assuming that one obstacle actually contained two mGFP molecules to account for dimers. This changes the *x*-axis coordinates corresponding to the determined *D*_ON_/*D*_OFF_ values—with a brightness value of a single mGFP of 500 per μm^2^ and a bulk brightness value of e.g., 50,000 per μm^2^, analysis assuming monomers would use a density of 1000 POI per μm^2^, analysis assuming dimers would use a density of 500 per μm^2^.

### Protein and Lipid Sizes Derived from Structural Data

To contextualize our experimentally determined apparent protein sizes *R*_POI,app_, we used available protein structural data to derive *R*_POI_, which is the radius of a hypothetical circular obstacle. For this, we extracted the positions of all atoms in the outer membrane leaflet and determined the cross-sectional area of a convex hull around their z-projection. We assumed every atom having the size defined by the van der Waals radius.^64^ Atom coordinates were obtained from an NMR structure (β2-AR monomer^32^ PDB ID: 6KR8) or a crystal structure (β2-AR dimer,^34^ PDB ID: 2RH1) ORAI (hexamer,^27^ PDB ID: 4HKR). Note that the crystal structure used for Orai1is in fact of the *D. melanogaster* ortholog, which shares ∼73% sequence homology with Orai1. All PDB files were downloaded from the Orientation of Proteins in Membranes (OPM) database,^65^ which provides spatial positions of membrane proteins in the lipid bilayer. The size of Orai1-mGFP hexamer was estimated based on a structure predicted with AlphaFold-multimer.^38,66^ For HA-WT and HA-Δpalm, the 3D structure of the transmembrane helix, the cytoplasmic tail and a truncated extracellular moiety (**GYK**DVILWFSFGASCFLLLAIAMGLVFICVKNGNMRCTICI) was predicted with AlphaFold2 ^38^ accessed via ColabFold,^67–70^ with the orientation in the membrane calculated via PPM 3.0.^39^

For the estimation of the minimal tracer size, we used the area per lipid of stearoyl sphingomyelin^30^ and calculated the radius. For the maximal size estimate, the area of the rigid triazole moiety was added, which is part of the headgroup region in the labeled sphingomyelin derivative. The triazole coordinates were obtained from ChemSpider (ChemSpider ID: 60839), hydrogens were manually added in the open-source platform Avogadro.

### Modeling Data to Assess Possible NERD Characteristics

To assess the effect that NERDs would have in our assay we used a previously established empirical model (eq 3) that describes tracer diffusion behavior in the presence of inert obstacles with coimmobilized NERDs.^24^

3 $$\frac{D_{\mathrm{ON}}}{D_{\mathrm{OFF}}}=\left(1-\frac{1-e^{-\rho_{\mathrm{POI}}\pi {\left(R_{\mathrm{POI}}+R_{\mathrm{lipid}}\right)}^{2}}}{C_{p}}\right)\cdot \left(1-\left(1-e^{-\rho_{\mathrm{POI}}\pi x}\right)\cdot \left(1-f_{\mathrm{NERD}}\right)+\left(e^{-2\rho_{\mathrm{POI}}\pi x}-e^{-\rho_{\mathrm{POI}}\pi x}\right)\cdot {\left(1-f_{\mathrm{NERD}}\right)}^{2}\right)$$

with *x* = (*R*_POI_ + *R*_lipid_ + *d*_NERD_)^2^ – (*R*_POI_ + *R*_lipid_)^2^

This model returns diffusion ratios *D*_ON_/*D*_OFF_ as a function of ρ_POI_, *R*_POI_, *R*_lipid_, *f*_NERD_, *d*_NERD_. Using estimates of *R*_POI_ and *R*_lipid_ as described above we modeled (ρ_POI_, *D*_ON_/*D*_OFF_) value pairs assuming different nanofeature sizes (*d*_NERD_ *)* and diffusivities (*f*_NERD_), taking also into account parameters and errors inherent to experimental data for a specific POI: A density value ρ_POI_ was drawn from the probability distribution of experimentally determined POI densities. The corresponding value for *D*_ON_/*D*_OFF_ was determined using eq 2. To account for experimental errors both the selected POI density as well as the calculated diffusion ratio were assumed as the center of a Gaussian distribution with a standard deviation corresponding to the respective relative experimental error. From these Gaussian distributions, (ρ_POI_, *D*_ON_/*D*_OFF_ *)* value pairs were randomly drawn. We also considered the size of the data set, i.e., the number of recorded cells for a particular POI. First, a large data pool of several thousand (ρ_POI_, *D*_ON_/*D*_OFF_) value pairs was generated as described above. Next, we performed 1000 iterations of randomly drawing *n* modeled value pairs from this data set, with *n* specifying the number of cells within the analyzed data set. In each iteration, drawn data points were fitted with eq 1, i.e., the same as used to fit experimental data, to arrive at a mean radius *R*_POI,app_ and the corresponding error δ*R*_POI,app_ thus creating the result that we would extract from experimental data when measuring a POI featuring a particular NERD with a given *d*_NERD_ and *f*_NERD_ (see Supporting Figure S7).

To establish the NERD properties that would be consistent with our data, we compared the experimentally determined *R*_POI_,_app_ values with the modeled ones. If the corresponding intervals *R*_POI,app_ ± δ*R*_POI,app_ did not overlap, the assumed NERD characteristics were classified as not consistent with our experimental data. In case the mean value *R*_POI,app_ was within the interval of the *d*_NERD_ = 0 scenario, the NERD was classified as borderline; if intervals overlapped, the NERD was classified as consistent (see Supporting Figure S7). These classifications were performed for all considered values of *f*_NERD_ and stored in matrices (see Figure 4B–F).

## Supplementary Material



## Notes

Exemplary mean square displacements over time (Figure S1); generation of selection masks and application to diffusion data (Figures S2 and S3); effect of obstacle ellipticity on the percolation threshold (Figure S4); POI size calculation from 3D structures (Figure S5); Structure and size of Orai1 hexamer including mGFPs (Figure S6); Using modeled data to determine possible characteristics of POI-associated nanoenvironments (Figure S7); correlation analysis between *D*_OFF_ and ρ_POI_ (Table S1); average number of localizations and trajectories per cell (Table S2) (PDF)
