# Supplementary material for: Quantitative Mapping of the Lipid Nanoenvironment around Transmembrane Proteins in Living Cells
Source: ACS Nano. 2026 Jan 10;20(3):3019–29. doi: 10.1021/acsnano.5c19300 (PMC12854754; doi:10.1021/acsnano.5c19300)
Supplement: Supplementary file 1 [file nn5c19300_si_001.pdf]

SUPPLEMENTARY INFORMATION

Quantitative mapping of the lipid nanoenvironment around transmembrane proteins in living cells

Veronika Brumovska[*,1], Marina Bishara[*,1], Andreas M. Arnold[2], Barbora Kalouskova[1], Gergö Fülöp[1], Marc Fahrner[3], Isabella Derler[3], Lena Maltan[3], Nobuaki Matsumori[4], Mario Brameshuber[1], Gerhard J. Schütz[1], Eva Sevcsik[1]

[*]contributed equally

[1] Institute of Applied Physics, TU Wien, Wiedner Hauptstrasse 8-10, 1040 Vienna, Austria

[2]Biochemistry & Biophysics Center, National Heart, Lung, and Blood Institute, National Institutes of Health, 50 South Drive, Bethesda 20892, MD, USA

[3]Institute of Biophysics, JKU Life Science Center, Johannes Kepler University Linz, Gruberstraße 40, A-4020 Linz, Austria

[4] Department of Chemistry, Graduate School of Science, Kyushu University, Fukuoka 819-0395, Japan

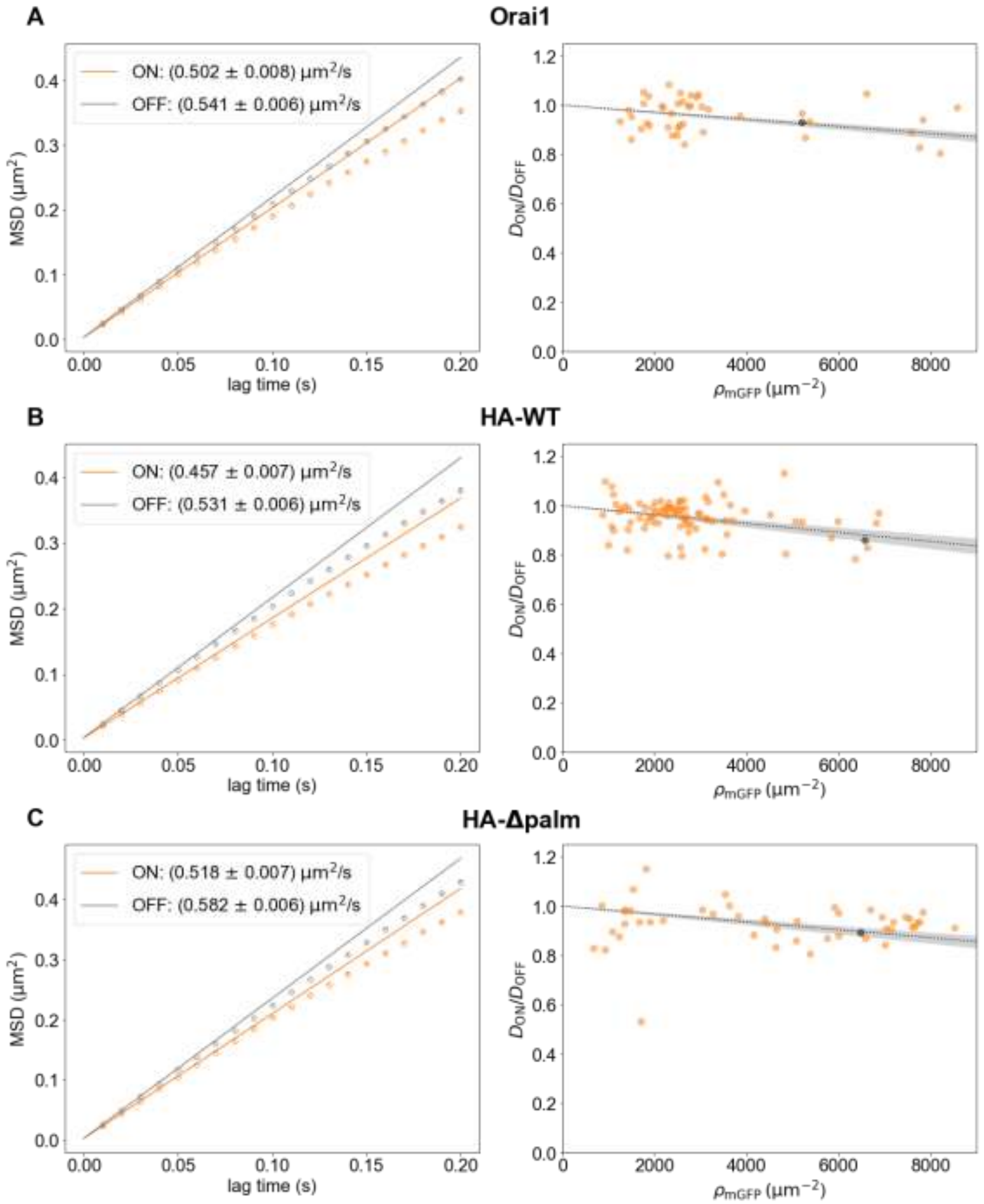

Supplementary Figure S1. Exemplary mean square displacements over time. Mean square displacement (msd) of the tracer molecule SM-Atto594 as a function of t_{lag} recorded in ON (orange) and OFF (grey) areas for a representative cell expressing mGFP-tagged Orai1 (n=43 cells) **(A)**, HA-WT (n=85 cells) **(B)** and HA-Δpalm (n=48cells) **(C)** (left). Relative diffusion coefficients D_{ON}/D_{OFF} were determined for individual cells, plotted as a function of POI density in ON areas, ρ_{POI}, and fitted with Eq. 1 (right). Fits are shown as black dotted lines with areas shaded in grey indicating fit errors. The grey dot in the images on the right indicates the particular cell shown on the left.

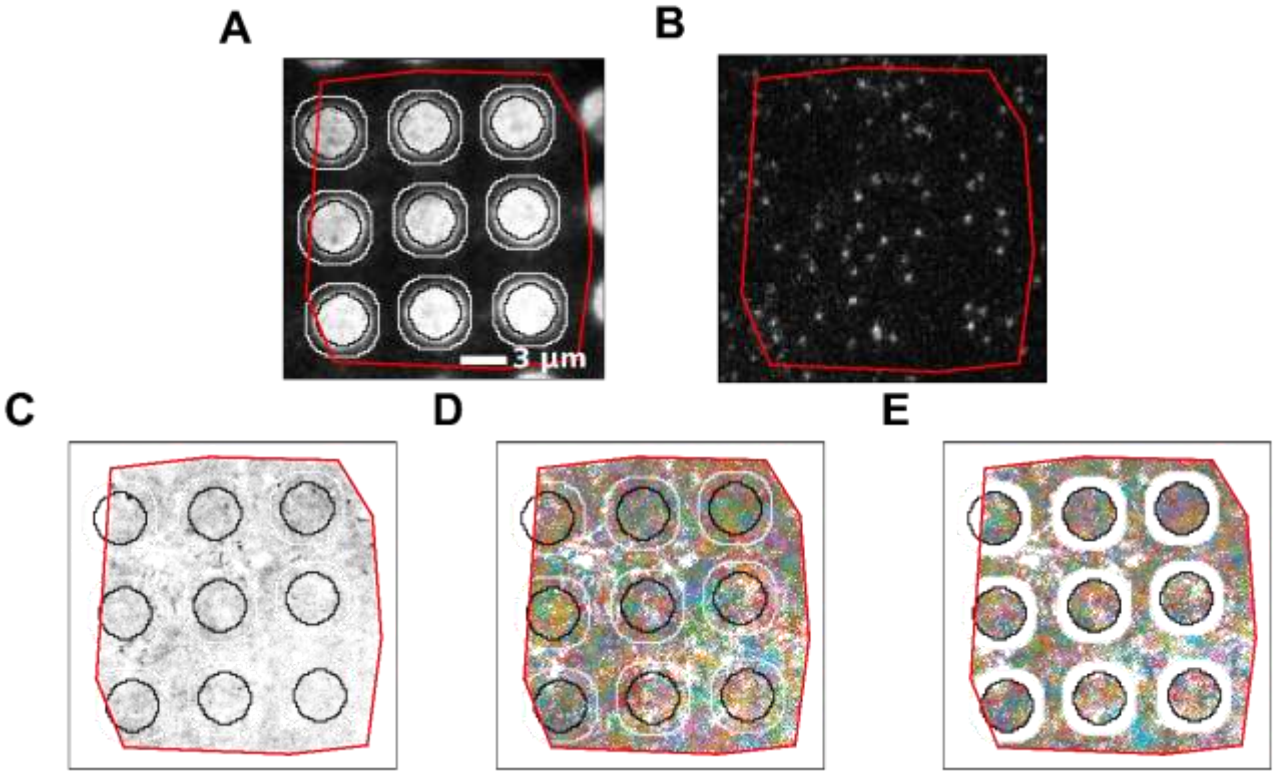

Supplementary Figure S2. Generation of selection masks and application to diffusion data. (A) Selection masks separating ON and OFF areas were constructed based on the mGFP pattern recorded in the blue color channel. The outlines of the mGFP patterns were selected using the brush selection tool in ImageJ. To exclude trajectories located at ON/OFF boundaries - where transitions between ON and OFF areas may occur - masks for ON (black circles) and OFF (white rounded square) areas were generated by dilating and eroding pattern edges, respectively. The analyzed region of interest is indicated in red. **(B)** Representative movie frame showing individual lipid tracer molecules, recorded in the yellow color channel. **(C)** Singe-molecule localizations in an exemplary cell, with each point representing one localization (typically 100,000-200,000 localizations per cell). **(D)** Localizations were linked to generate single-molecule trajectories (typically 2,000-3,000 trajectories per cell), which were assigned to ON and OFF areas according to the selection mask for subsequent diffusion analysis **(E)**.

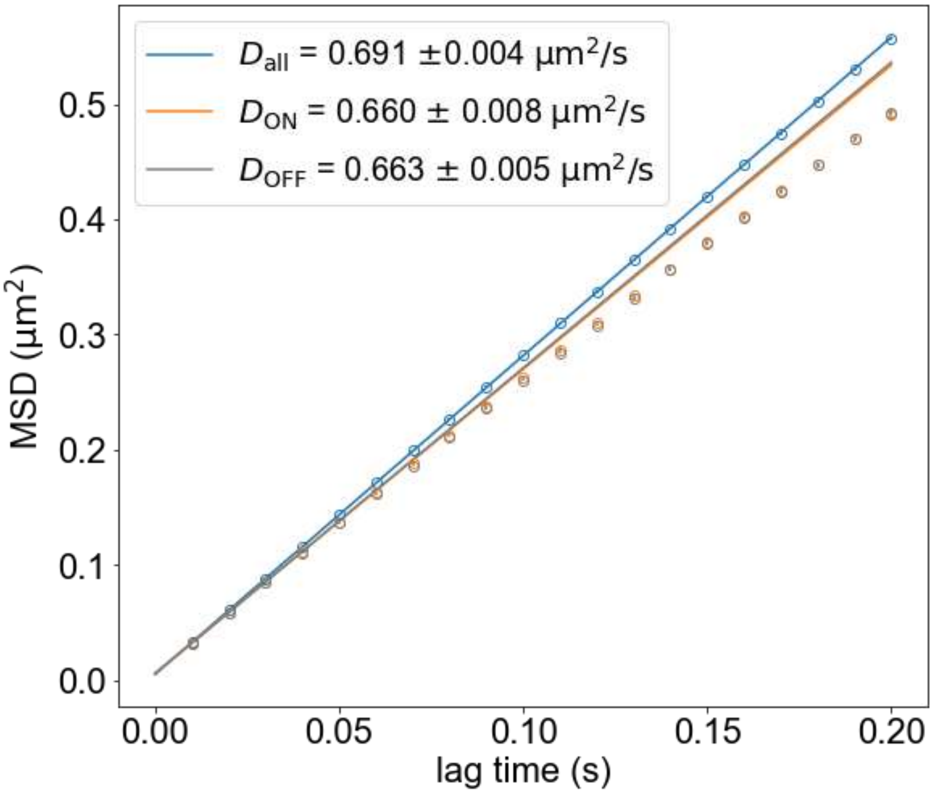

Supplementary Figure S3. Application of circular selection masks does not affect the determined diffusion coefficient in non-patterned cells. A circular mask used in micropatterning experiments (~ 3 µm diameter, 3 µm interspace; for details see Methods section) was applied to diffusion data recorded in a non-patterned cell expressing β2-AR. The deviation from linearity at lag times above ~50-100 ms is a consequence of the selection mask (see Figure S2), as it introduces a confinement area: our analysis only includes trajectories which are fully included in either ON or OFF areas, and discards trajectories showing transitions between the areas. As it is more likely that such transitions occur in trajectories with long end-to-end distance, they are absent in the analyzed statistical sample, resulting in apparent subdiffusion behavior. Resulting ON (D_{ON}) and OFF (D_{OFF}) diffusion constants are in good agreement with the diffusion constant derived from all trajectories (D_{all}).

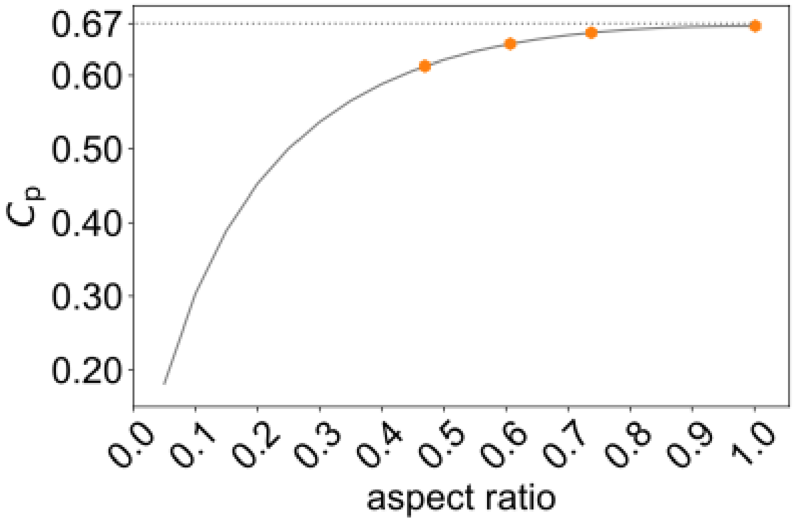

Supplementary Figure S4. Effect of obstacle ellipticity on the percolation threshold. The theoretical frameworks underlying our simulations are based on the area coverage by the obstacle, with the percolation threshold (C_P) as th

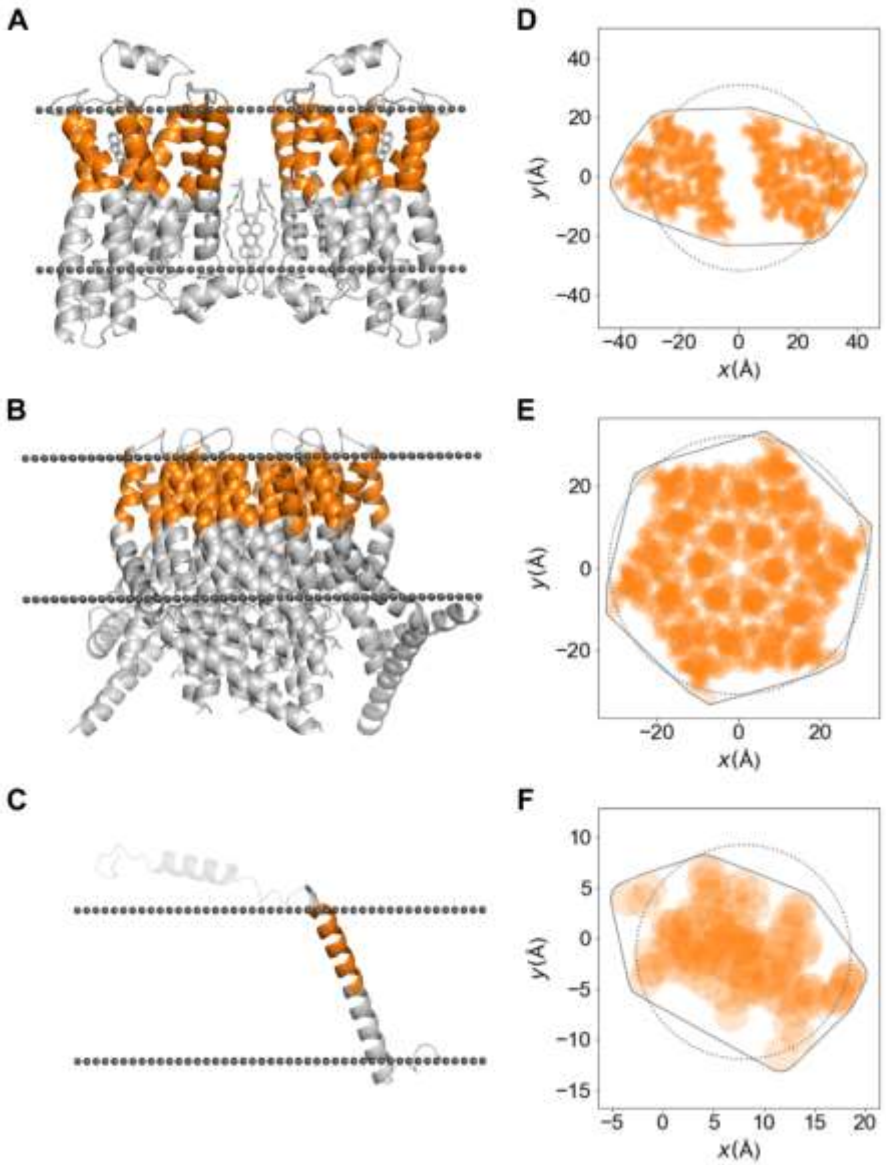

Supplementary

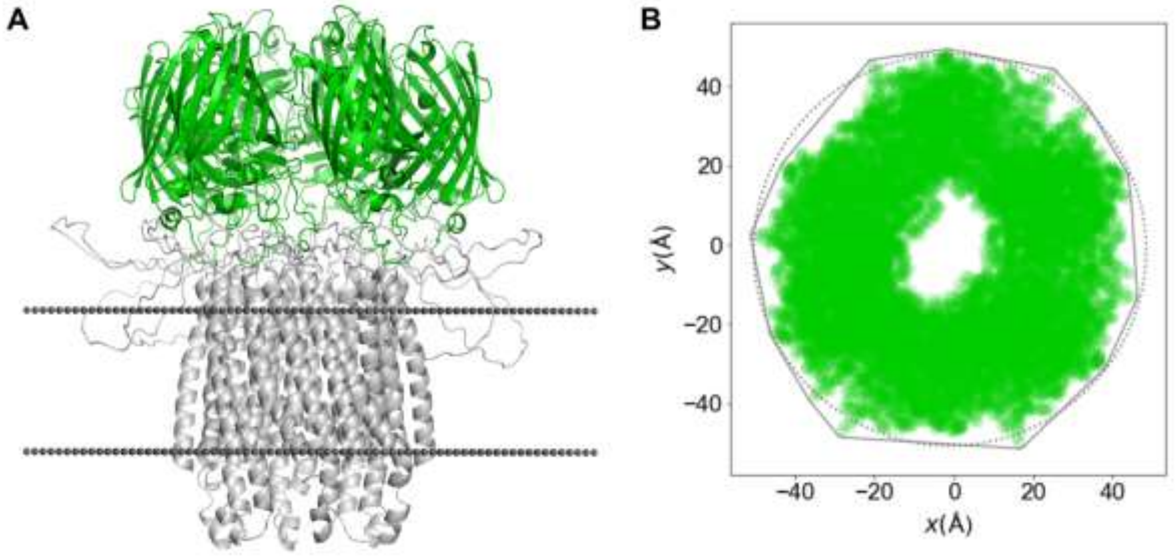

Supplementary Figure S6. Structure and size of Orai1 hexamer including mGFPs. (A) 3D structure of

h

e

x

a

m

e

Note on extracellular contributions to R_{POI}

r

The extracellular part of the lipid tracer consists of the fluorescent label Atto594 (diameter ~ 0.9 nm) on a short

i

P

c

E

G

O

9

r

a

l

i

i

1

n

k

w

e

i

r

t

h

(

c

a

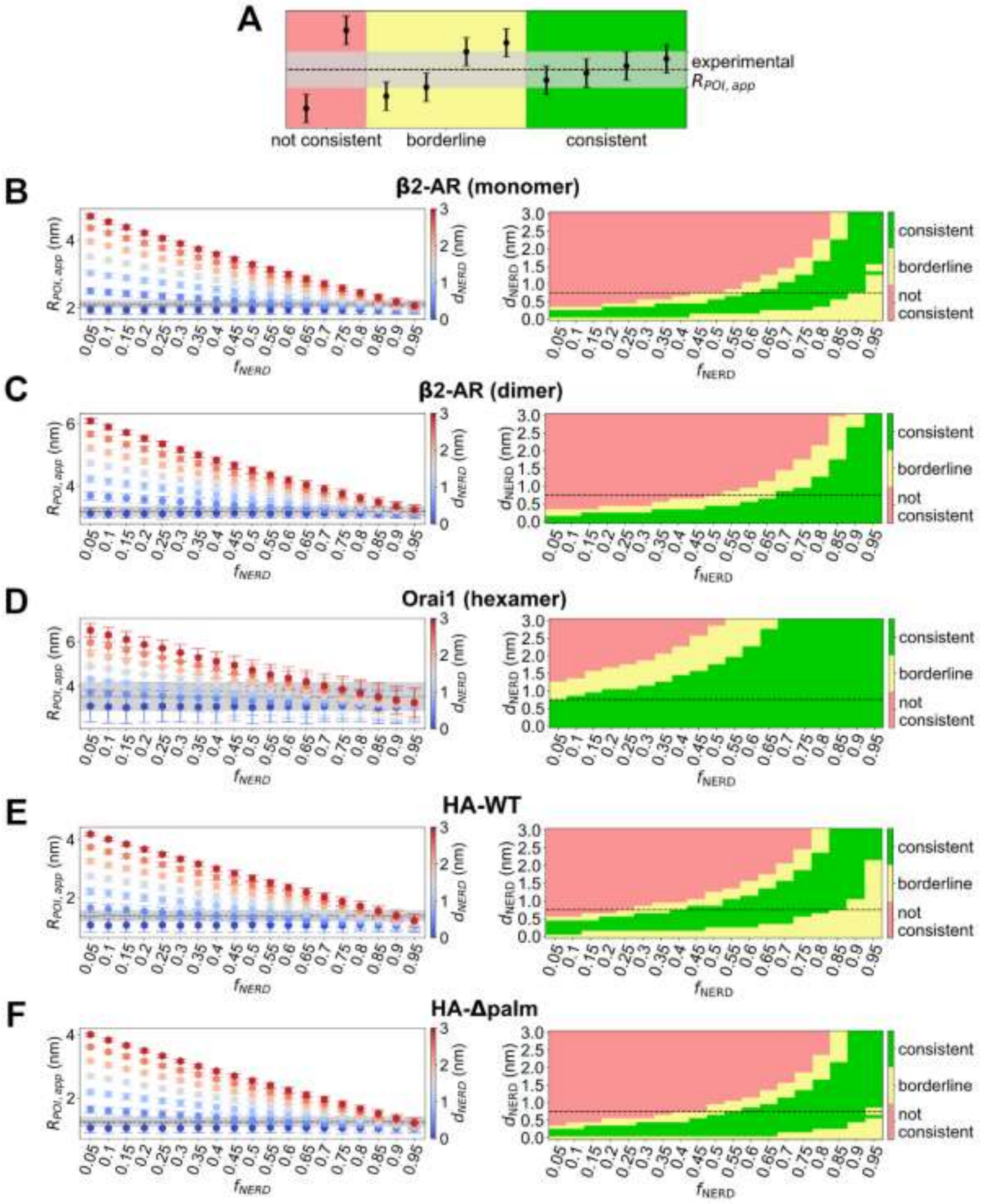

Supplementary Figure S7. Using modeled data to determine possible characteristics of POI-associated nano-environments. Immobilized proteins with R_{POI} are assumed to be surrounded by NERDs with a width d_{NERD}, where tracer mobility is reduced by a factor f_{NERD} compared to the bulk membrane (OFF) area. Applying Eq. 2 yielded $R_{\mathrm{POI,app}}$ values for in total 133 scenarios with different d_{NERD} and f_{NERD}. **(A)** If the intervals $R_{\mathrm{POI,app}} \pm \delta R_{\mathrm{POI,app}}$ from experiment and simulation did not overlap, the NERD characteristics were classified as not

consistent with our experimental data (light red). In case the mean value $R_{POI,app}$ was within the interval of the $d_{NERD} = 0$ scenario, NERD characteristics were classified as borderline (yellow) or, if intervals did overlap, as consistent (green). Results are shown for **(B)** β2-AR (monomer), **(C)** β2-AR (dimer), **(D)** Orai1 (hexamer), **(E)** HA-WT and **(F)** HA-Δpalm. The black dashed line in the left plot indicates the experimentally determined $R_{POI,app}$ value; the grey shaded area indicates the associated error. Scenarios below the dashed line correspond to values of dNERD below 0.7 nm, corresponding to a single layer of lipids.

Supplementary Tables:

Supplementary Table S1: Pearson correlation analysis reveals no correlation between the diffusion coefficient of SM-Atto594 in OFF areas (D_{OFF}) and the density of immobilized protein in ON areas, ρ_{POI}, in patterned cells.

	**Pearson correlation coefficient, r**	**p-value, p**	**null hypothesis (no correlation) rejected, p < 0.05**
**β2-AR**	0.097709418	0.365116131	FALSE
**HA-WT**	-0.052684393	0.632033208	FALSE
**HA-Δpalm**	0.109957736	0.456883242	FALSE
**Orai1**	0.234391964	0.130309146	FALSE

Supplementary Table S2: Average number of localizations and trajectories per cell

Average number of localizations per cell						
	total	SEM	ON	SEM	OFF	SEM
**β2-AR**						
**HA-WT**						
**HA-Δpalm**						
**Orai1**						
**Average number of trajectories per cell**						
**β2-AR**						
**HA-WT**						
**HA-Δpalm**						
**Orai1**						

References

(1) Xia, W.; Thorpe, M. F. Percolation Properties of Random Ellipses. *Phys. Rev. A* **1988**, *38* (5), 2650–2656. https://doi.org/10.1103/physreva.38.2650.

(2) Cherezov, V.; Rosenbaum, D. M.; Hanson, M. A.; Rasmussen, S. G. F.; Thian, F. S.; Kobilka, T. S.; Choi, H.-J.; Kuhn, P.; Weis, W. I.; Kobilka, B. K.; Stevens, R. C. High-Resolution Crystal Structure of an Engineered Human B2-Adrenergic G Protein–Coupled Receptor. *Science* **2007**, *318* (5854), 1258–1265. https://doi.org/10.1126/science.1150577.

(3) Hou, X.; Pedi, L.; Diver, M. M.; Long, S. B. Crystal Structure of the Calcium Release–Activated Calcium Channel Orai. *Science* **2012**, *338* (6112), 1308–1313. https://doi.org/10.1126/science.1228757.

(4) Jumper, J.; Evans, R.; Pritzel, A.; Green, T.; Figurnov, M.; Ronneberger, O.; Tunyasuvunakool, K.; Bates, R.; Žídek, A.; Potapenko, A.; Bridgland, A.; Meyer, C.; Kohl, S. A. A.; Ballard, A. J.; Cowie, A.; Romera-Paredes, B.; Nikolov, S.; Jain, R.; Adler, J.; Back, T.; Petersen, S.; Reiman, D.; Clancy, E.; Zielinski, M.; Steinegger, M.; Pacholska, M.; Berghammer, T.; Bodenstein, S.; Silver, D.; Vinyals, O.; Senior, A. W.; Kavukcuoglu, K.; Kohli, P.; Hassabis, D. Highly Accurate Protein Structure Prediction with AlphaFold. *Nature* **2021**, *596* (7873), 583–589. https://doi.org/10.1038/s41586-021-03819-2.

(5) Mirdita, M.; Schütze, K.; Moriwaki, Y.; Heo, L.; Ovchinnikov, S.; Steinegger, M. ColabFold: Making Protein Folding Accessible to All. *Nat. Methods* **2022**, *19* (6), 679–682. https://doi.org/10.1038/s41592-022-01488-1.

(6) Mirdita, M.; Steinegger, M.; Söding, J. MMseqs2 Desktop and Local Web Server App for Fast, Interactive Sequence Searches. *Bioinformatics* **2019**, *35* (16), 2856–2858. https://doi.org/10.1093/bioinformatics/bty1057.

(7) Mirdita, M.; von den Driesch, L.; Galiez, C.; Martin, M. J.; Söding, J.; Steinegger, M. Uniclust Databases of Clustered and Deeply Annotated Protein Sequences and Alignments. *Nucleic Acids Res.* **2017**, *45* (D1), D170–D176. https://doi.org/10.1093/nar/gkw1081.

(8) Evans, R.; O'Neill, M.; Pritzel, A.; Antropova, N.; Senior, A.; Green, T.; Žídek, A.; Bates, R.; Blackwell, S.; Yim, J.; Ronneberger, O.; Bodenstein, S.; Zielinski, M.; Bridgland, A.; Potapenko, A.; Cowie, A.; Tunyasuvunakool, K.; Jain, R.; Clancy, E.; Kohli, P.; Jumper, J.; Hassabis, D. Protein Complex Prediction with AlphaFold-Multimer. *bioRxiv* **2022**, 2021.10.04.463034. https://doi.org/10.1101/2021.10.04.463034.

(9) Lomize, A. L.; Todd, S. C.; Pogozheva, I. D. Spatial Arrangement of Proteins in Planar and Curved Membranes by PPM 3.0. *Protein Sci* **2022**, *31* (1), 209–220. https://doi.org/10.1002/pro.4219.

(10) Ma, Z.; LeBard, D. N.; Loverde, S. M.; Sharp, K. A.; Klein, M. L.; Discher, D. E.; Finkel, T. H. TCR Triggering by PMHC Ligands Tethered on Surfaces via Poly(Ethylene Glycol) Depends on Polymer Length. *Plos One* **2014**, *9* (11), e112292. https://doi.org/10.1371/journal.pone.0112292.

(11) Hink, M. A.; Griep, R. A.; Borst, J. W.; Hoek, A. van; Eppink, M. H. M.; Schots, A.; Visser, A. J. W. G. Structural Dynamics of Green Fluorescent Protein Alone and Fused with a Single Chain Fv Protein*. *J Biol Chem* **2000**, *275* (23), 17556–17560. https://doi.org/10.1074/jbc.m001348200.
